# Innoculation for Pleuro-Pueumonia

**Published:** 1883-10

**Authors:** 


INOCULATION FOR PLEURO-PNEUMONIA.—Dr. Ezra M. Hunt, Secretary of the State Board of Health of New Jersey, induced the Legislature to permit inoculation for pleuro-pneumonia under certain restrictions.

The Board proposed the inoculation as a settled principle approved by such eminent authorities as Willems of Belgium, Rutherford of Edinburgh, and Fleming of London, the opinion of these gentlemen being that when properly performed inoculation could not communicate the disease.

The results so far tend to prove the value of inoculation as affording immunity against the propagation of the infection.

